# California State Veterinary Medical Association

**Published:** 1894-09

**Authors:** R. A. Archibald

**Affiliations:** Secretary; Sacramento, Cal.


### CALIFORNIA STATE VETERINARY MEDICAL ASSOCIATION.

The California State Veterinary Medical Association held its regular quarterly meeting at the Baldwin Hotel, San Francisco, Cal., on March 14th, 1894.

The meeting was called to order by the President, Dr. H. A. Spencer. Owing to the absence of the secretary, Dr. D. F. Fox was appointed secretary pro tem. Upon roll call the following named gentlemen answered to their names:—Drs. Maclay, Spencer, Sr., Spencer, Jr., Wadams, Orvis, Pierce, Robin, Williams, Forrest, Jackson, Hogarty, Eddy and Fox; visitor, I. B. Dalziel.

On motion by *Dr. Maclay*, the reading of the minutes of the previous meeting was dispensed with, and they were adopted.

*The President* then delivered his inaugural address, saying:

Gentlemen:—At the commencement of the year, by your selection, I became your presiding officer, a compliment to myself that, in my somewhat limited vocabulary, I am unable to glean words adequate to convey my gratitude. I shall endeavor, if careful study and strict attention to the business of your deliberations profit anything, to work zealously for the interests of not only this Association, but for that of the entire profession. How well I shall succeed depends in a great measure upon the sympathy and assistance you may accord me; a hearty co-operation in the maintenance of order, in securing a reputable attendance, and a cheerful response to the appointment of essayists, is the desideratum, and without which even the brightest lights in the Association would fail. It would be the most gross egotism for me to aspire to that perfection as counsellor and parliamentarian that has successfully attended the incumbences of my learned predecessors, those gentlemen chosen for their peculiar aptitude to direct the affairs of this organization through its infancy, when it was endangered by shoals and adverse currents on every hand. And if I succeed in winning your toleration during the ensuing year, and can transfer to my successor a larger roll of attending members, and a better financial condition than we now possess, I shall have realized my most sanguine expectations.

In addressing you upon this occasion, I have deemed it best to offer my idea of what a veterinarian, both socially and professionally, should be.

To my notion a veterinarian should possess all the attributes of a gentleman, his education should be of a high order, he should be devoted to his profession, ever analyzing new theories, eagerly delving for knowledge that may be profitably applied to the amelioration of the ills and injuries of such patients as he may be summoned to attend. It is his duty to strictly adhere to the ethics of his profession, always bearing in mind that through their mediumship only can the dignity of his avocation be sustained. He should cultivate a sympathetic feeling for those unfortunates that the merciful owner seeks his services to relieve; nothing so thoroughly disgusts a client as unnecessary infliction of pain or a rough, coarse tone or manner. Honesty to patrons is a policy that none can afford to forego, for while we may secure a valuable fee by deception, we will most certainly incur a suspicion that will not stop with the aggrieved party.

Ours is a profession acquired by close application extending over a number of years, varying in period according to the curriculum that the several schools or colleges may dictate, and he that would be successful must acquit himself of the intention to amass more than a simple competency. He should espouse the profession of a veterinarian not to get wealthy; but for the higher and nobler reason, the love of the cause. There is no employment among all the vocations of life more grand, more honorable or commendable than that of the individual who, from love of the creatures given to man for his use and companionship, has, by arduous study, keen sympathy and energy, prepared himself for cure or amelioration of their infirmities, and to curb the propensities that many of their diseases have to attack the life and health of the human race.

The science of veterinary medicine and surgery comprises a knowledge of the structure, anatomy and physiology of the economy of the domesticated animal, with an intelligent, discriminating cognition of their pathological symptoms and treatment, their relationship when diseased with regard to the r communicability of the same to mankind, hence an intelligent understanding and application of bacteriology must prove of inestimable importance in respect to checking the ravages of infectious and contagious diseases, and should be a part of the veterinarian's stock in trade, to whose vigilance as a sanitarian the world must ultimately acknowledge a debt of gratitude.

A year has now passed since, through the almost superhuman exertions of this Association, an enactment for the partial protection of veterinary medicine and surgery was piloted through the windings and sinuosities of the Senate and Assembly of the State of California. It is needless to recount the trials, tribulations and disappointments our members, and especially those of the Legislative Committee, were subjected to in their efforts to inculcate into the "think tank" of the average California law maker that we sought only to attain that distinction, recognition and protection that our usefulness and education proclaimed us entitled to, and that in giving us our right they were subserving the interests of the commonwealth; but suffice to remind you that only after arduous and unceasing devotion to the sessions of three legislations your committee, with *your very hearty co-operation*, succeeded in getting a statutory enactment passed and signed by the Governor, that, while it is restricted in its usefulness to certain conditions that in a measure impair its effectiveness, it is acknowledged to be the best law of like nature in the United States, and affords a very good foundation for subsequent amendments. It dictates a distinction between the veterinarian and the empiric that the public are by no means slow in appreciating. This is demonstrated in the fact that now the former's services are sought for in nearly all instances where a professional attendance is necessary; while on the other hand the quack is continually reminded of the fact that legally and morally he is restrained from practice, and can only participate in the same by evasion; he is only tolerated, not patronized. These reminders place him in the very unenviable position that, unless he be devoid of pride, he will seek a more congenial and lucrative occupation. But while all this is gratifying, we must still be alive to the necessity of concerted action. Our work is only fairly begun. In addition to a more stringent and far-reaching law, we must have sanitary enactments, quarantine regulations, and above all, an officer to see that these shall be effective. Nor will it do for us to confidently rely on our own ability to carry these measures to a successful issue; we must secure the assistance of every available influence. The Press must be solicited to mould opinions and educate the public to the necessity of meting out justice to an honored profession. The medical profession, who though dilatory, are finally realizing our importance as co-operators, in the great work of protecting humanity from the inroads of pestilence and disease, and contributors, plying our avocation to the promotion and longevity of mankind, and by their sympathy and support much can be accomplished toward the final successful termination of our rights. A dignified and courteous demeanor, a studied regard for veterinary ethics, sandwiched with determined honesty, must certainly tend more to give us the social status that secures prestige and begets respect. And while I have no desire to arraign the members of our profession, still these attributes to gentility will go far toward elevating the veterinary profession to a standard which will place it on an equal footing with its sister profession.

Let me earnestly appeal to you to lend your support and sympathy to the United States Veterinary Medical Association, through whose efforts we must look for further elevation of our profession, and by whose assistance only can we look for higher matriculations and more extended curriculums in our colleges. Through its efforts we will receive better recognition, more remunerative positions, and distinction from the Government.

And finally, I beseech you to give your hearty support, individually and collectively, to the welfare of our own organization. Let each of us resolve to be in the van of progression, and laying aside our jealousies, prejudices and business differences, to make the California State Veterinary Medical Association for the promulgation of knowledge and social intercourse.

*Dr. Spencer* then read a paper entitled Purpura Hemorrhagica, Oedema, Anasarca, etc. (vide fol. 245).

The discussion was opened by *Dr. Maclay*. He highly recommended the use of cold applications to the swellings. He said there was no similarity between the subject under condsideration and lymphangitis. The subject was further discussed by Drs. Orvis and Forrest.

*Dr. Pierce* presented a paper entitled Parturient Apoplexy (vide fol 248).

The discussion was opened by *Dr. Wadams*, who recommended for treatment croton oil and stimulants *Dr. Spencer, Jr.*, also recommended stimulants. *Dr. Orvis* suggested that different treatments should be given to different veterinarians and let them try them and report to the Association. His idea of treatment is hyposulphite of soda and injections of stimulants, as alcohol, nux vomica, etc. *Dr. Jackson* has been very successful with the use of nux vomica and belladonna followed two days after by purgatives and enemas. *Dr. Pierce* said he had used sufphate of eserine with success. *Dr. Forrest* also favored the use of stimulants. *Dr. Maclay* spoke on the pathology of the disease, he thought the vascular system should receive more notice than the nervous system. *Dr. Spencer, Jr.*, was of the opinion that the disease was not apoplexy. *Dr. Maclay* said he heartily agreed with Dr. Spencer. There was considerable more discussion on this point, which all tended to show that all of the members had reached the conclusion that the term apoplexy was nothing more than an "asylum ignorantæ."

The president then closed the discussion with a few well chosen remarks.

As Dr. Archibald was not present, his paper was read by Dr. Fox, the subject being, "The Sympathetic nerve System, or why Orificial Surgery should cure or affect most Chronic Diseases, explained from an Anatomical and Physiological Basis." The paper was well received, and was discussed by most of the members present.

The President, in closing the discussion, mentioned a number of cases where he had seen good results from the application of orificial surgery in the human family.

Under the head of new business, Dr. Maclay moved that a vote of thanks be extended to the essayists for the able and masterly manner in which they had entertained the meeting. The motion was seconded by Dr. Orvis, and was carried.

On motion by Dr. Maclay, the Secretary was instructed to forward the results of the meeting to the veterinary journals.

On motion by Dr. Maclay, a vote of thanks was extended to Drs. Archibald and Fox for the manner in which they had entertained the members at the last annual meeting in Sacramento.

The subject matter of the lost certificates was brought up and discussed, whereupon on motion by Maclay, the President was requested to appoint a committee to look into the matter. The President appointed Drs. Orvis and Robin. Dr. Orvis requested that Dr. Spencer, Jr. be substituted in his place, as it was impossible for him to attend to the matter. There being no oblections, it was so ordered.

Dr. Maclay then spoke at some length on the good of the Association, also on legislative matters.

Dr. Fox moved that the President appoint a committee of five on legislative matters, and that the committee be empowered to represent the Association at the meeting of the State Sanitary Convention in San Jose next month, also that the President appoint himself as one of the committee. The motion was seconded by Dr. Pierce and carried.

The President then appointed the following gentlemen on the Legislative Committee, viz., Drs. Maclay, Spencer, Sr., Archibald, Orvis and Skaife.

The President appointed the following named gentlemen as essayists for the next meeting, viz. Drs. Fox, Eddy and Forrest.

There being no further bnsiness before the meeting, it adjoined to meet in San Francisco, June 13th, 1894.

R. A. ARCHIBALD, D.V.S., Secretary.

SACRAMENTO, CAL.

